# Ku80 correlates with neoadjuvant chemotherapy resistance in human lung adenocarcinoma, but reduces cisplatin/pemetrexed-induced apoptosis in A549 cells

**DOI:** 10.1186/s12931-017-0545-6

**Published:** 2017-04-11

**Authors:** Bin Shang, Yang Jia, Gang Chen, Zhou Wang

**Affiliations:** Department of Thoracic Surgery, Shandong Provincial Hospital Affiliated to Shandong University, 324 Jingwu Road, Jinan, Shandong 250021 China

**Keywords:** Ku80, Lung adenocarcinoma, Neoadjuvant chemotherapy, Chemoresistance

## Abstract

**Background:**

Ku80 is a DNA repair protein which involves in cell apoptosis and chemoresistance. However, it is unclear whether Ku80 correlates with the efficiency of neoadjuvant chemotherapy in human lung adenocarcinoma, and modulates cisplatin/pemetrexed-induced lung cancer cell apoptosis in vitro.

**Methods:**

We recruited 110 patients with stage IIIA lung adenocarcinoma, who received 2 cycles of neoadjuvant chemotherapy, and their lungs were reevaluated by CT scan. Immunohistochemistry and qRT-PCR was performed to detect the expression level of Ku80. A549 cells were transfected by lentiviral vector containing shRNA and full length cDNA to knockdown or upregulate Ku80 gene expression. CCK8 assay, flow cytometry and Western blot were employed to determine the viability and apoptosis of A549 cells treated with cisplatin combined with pemetrexed.

**Results:**

Ku80 expression was detected in 76 patients (69%). There were 38 patients who responded to chemotherapy, where Ku80 was positively expressed in 7 cases (18.4%). Immunohistochemical score of Ku80 protein in the response group (2.079 ± 1.617) to chemotherapy was lower than that in the nonresponse group (5.597 ± 2.114, *P* < 0.05). Tissue samples from the nonresponse group exhibited higher Ku80 mRNA levels compared to the response group. Ku80 knockdown by shRNA augmented cisplatin/pemetrexed-induced decline in viability, whereas Ku80 overexpression attenuated viability reduction induced by these drugs compared to control A549 cells. Both flow cytometry and Western blot analysis displayed that the apoptotic rate of Ku80 shRNA-transfected A549 cells was significantly increased compared to control cells treated with cisplatin/pemetrexed, which was lowered by Ku80 overexpression.

**Conclusion:**

Ku80 could predict the probability of resistance to neoadjuvant chemotherapy in lung adenocarcinoma, and reduced cisplatin and pemetrexed-induced apoptosis in A549 cells.

## Background

Lung cancer is one of the top leading causes of death among all malignant cancers worldwide, while lung adenocarcinoma becoming most common histological subtype of lung tumor [1]. When patients are diagnosed as lung cancer, most of them has been at the middle-late stage, according to the tumor-node-metastasis (TNM) classification for non-small cell lung cancer (NSCLC) of UICC in 2009 [2]. For the stage (N2) of NSCLC patients, the prognosis of only curative resection remains unsatisfactory due to incomplete resection [3]. Curative surgery could be effective until tumor regresses apparently and ipsilateral mediastinal metastatic lymph nodes disappear after 2 cycles of chemotherapy. Therefore, the routine preoperative neoadjuvant radiotherapy or chemotherapy has been widely accepted. Platinum combines pemetrexed as the first-line neoadjuvant chemotherapeutic agents for lung adenocarcinoma [4]. However, when reevaluating the tumor status through contrast Computed Tomography (CT) after 2 cycles of neoadjuvant chemotherapy, we find that only a few patients can obtain ideal efficiency. Most patients develop chemoresistance, which leads to the poor therapeutic effect [5]. Therefore, finding an indicator to predict the resistance to neoadjuvant chemotherapy would promote our understanding of individual treatment strategy.

The heterodimeric Ku protein plays a significant role in DNA double-strand breaks (DSBs) repair, which contains two subunits of 70 kDa (Ku70) and 80 kDa (Ku80 or Ku86). It has been reported that Ku proteins bind free DNA termini of DNA protein kinase (DNA-PK) and activate the latter [6–8]. Ku80 protects against cisplatin-induced apoptosis for adenocarcinoma cells. Targeted knockdown of Ku80 enhances radiosensitivity and chemosensitivity [1]. However, most of studies are based on resection specimen after the operation. It is unknown whether Ku80 is associated with chemosensitivity for cisplatin combined with pemetrexed before the neoadjuvant chemotherapy initiation in lung adenocarcinoma.

In this study, Ku80 protein abundance in tissue samples obtained through fiberoptic bronchoscopy examination from 110 patients with lung adenocarcinoma was determined by immunohistochemistry. Based on the expression levels of Ku80 by immunohistochemistry, we divided patients into two groups (i.e., positive and negative). In these two groups of patients, we reevaluated tumor regression status after 2 cycles of neoadjuvant chemotherapy to determine tumor chemosensitivity to these drugs. We also employed lung adenocarcinoma A549 cells with manipulation of Ku80 gene expression so as to determine its role in regulating the chemosensitivity in terms of apoptosis in vitro.

## Methods

### Patients

Lung cancer patients consulted in the Department of Thoracic Surgery, Provincial Hospital Affiliated to Shandong University from September 2013 to September 2016 were examined by bronchoscopy. 110 patients with pathological diagnosis as lung adenocarcinoma with clinical classification stage IIIA were recruited for this study. No patient received chemotherapy, radiotherapy or gene-targeted treatments. All patients and their relatives provided the written informed consent, and the research was approved by the Ethical Committee of our institution. Tumor tissues were collected through bronchoscopic biopsy. After immunohistochemistry, patients were divided into two groups according to the Ku80 protein abundance (high and low expression). Two groups received same first-line neoadjuvant chemotherapy, with cisplatin 75 mg/m^2^ combined with pemetrexed 500 mg/m^2^ adjusted in terms of their creatinine clearance [9].

### Immunohistochemistry

Tissues were fixed by 4% paraformaldehyde overnight, embedded in paraffin and cut in 5 μm for experiments. Tissue sections were deparaffinized, hydrated, and heated in a steamer for antigen retrieval. The tissue samples were then incubated overnight with rabbit anti-human Ku80 monoclonal antibody (Thermo Fisher Scientific, Fremont, CA, USA) at 1:500 dilution, followed by incubation with goat anti-rabbit secondary antibody (Thermo Fisher Scientific, Fremont, CA, USA). The sections were washed extensively and incubated with DAB Quanto (Thermo Fisher Scientific, Fremont, CA, USA) for 5 min. The sections were stained, fixed, and visualized. Each section was examined by two pathological experts independently who were blinded to the clinical data. The immunohistochemical score (IHS) was obtained by combination of proportion score (average percentage of the five fields) with intensity score of the stained tumor cells. Staining intensity was divided into 3 level: 0 (negative), 1 (weak staining), 2 (moderate staining) and 3 (strong staining). The proportion of the tumor cells were as follow: 0 = 0 ~ 5%, 1 = 5 ~ 10%, 2 = 11 ~ 49% and 3 ≥ 50%. The final IHS ranged from 4 to 9 was considered positive staining [10].

### Real-time fluorescence quantitative PCR

Total RNA was extracted by using Trizol reagent (Invitrogen, USA) from tumor samples, and was subjected to reverse transcribed to cDNA according to manufacture’ instruction. M-MLV reverse transcriptase (Takara, Otsu, Japan) was used for cDNA synthesis. Real-time PCR reaction was proceeded using SyBR-Green in a LightCycler ® 480 Real-Time PCR system (Roche Diagnostics, Indianapolis, IN, USA). The primers sequences for Ku80 were (F) 5′-ACGATTTGGTACAGATGGCACTc-3′, (R) 5′-GCTCCTTGAAGACGCACAGTTT-3′. The β-actin primer sequences used were (F) 5′-TGGAGAAAATCTGGCACCAC-3′, (R) 5′-GGTCTCAAACATGATCTGG-3′. The relative mRNA expression was normalized to amplification of β-actin gene.

### Therapeutic evaluation

Chest CT scan was used to evaluate the response to chemotherapy after 2 cycles of therapy protocol. The result of therapy was classified by RECIST guideline (Version1.1) [11]. Complete Response (CR) was defined as all target lesions disappeared. The short axis of any enlarged lymph nodes must have reduction to <10 mm. Partial Response (PR) was defined as the sum of lesions diameters decreased at least 30 percentages. When either of these criteria met, this patient was classified in response group.

### Cell culture

Lung adenocarcinoma A549 cell line was purchased from Academia Sinica (Shanghai, China). Cells were growing as a monolayer in cell culture flasks containing Roswell Park Memorial Institute (RPMI) 1640 medium enriched with 10% fetal bovine serum (FBS) and antibiotics (1% penicillin/streptomycin) maintained in humidified incubators with 5% CO_2_ at 37 °C. Cells with logarithmic growth phase were used for experiment.

### Transfection by lentivirus

To knock down Ku80 expression, A549 cells were transfected with lentiviral vector including specific shRNA (specific sequence was: 5′-CTTTAACAACTTCCTGAAA-3′) and scrambled shRNA lentivirus (A549kd and NCkd, respectively). Meanwhile, to upregulate Ku80 gene expression, A549 cells were infected with lentiviruses contained full-length cDNA of Ku80 and the control lentiviral vector (A540oe and NCoe, respectively). The lentiviral vector green fluorescent protein (GFP) expressed in all lentiviruses was used to evaluate the transduction efficiency. All lentiviral vectors were purchased from the Genechem (Shanghai, China). The lentivirus transfection was performed in A549 cell line with MOI of 20:1 according to the manufacture’s instruction supplemented with 6 μg/mL polybrene. After 24 h, replacing medium containing vector by complete medium.

### Western blot

Protein of cells was extracted with RIPA lysis buffer plus phenylmethysulfonyl fluoride (PMSF) (Zhongshan Biotech, China) according to the manufacturer’s instruction. The protein concentration was measured by the Bio-Rad protein assay reagent (Bio-Rad). 40 μg of protein were mixed with 4x loading Buffer (Beyotime, China), which was loaded into each lane of 10% poly-acrylamide gel electrophoresis. Protein samples were transferred to PVDF membranes after electrophoresis. Membranes were blocked by incubation in TBS containing 0.1% Tween-20, supplement with 5% skim milk. PVDF membranes were then incubated overnight at 4 °C with mouse anti-human Ku80 (1:1,000 dilution, monoclonal; Abcam, Cambridge, UK), cleaved caspase 3 (1:1,000, polyclonal; Cell Signaling Technology, Bedford, MA, USA) or β-actin (1:10,000, monoclonal; Santa Cruz Biotechnology, Santa Cruz, CA, USA) antibodies. The membranes were then incubated with goat anti-mouse secondary antibody conjugated with horseradish peroxidase (HRP) (1:5000, Zhongshan Biotech, China) for 1 h at room temperature after washing by TBST. Protein bands were visualized using enhanced chemiluminescence (ECL) detection system LAS-4000 MINI System (GE, USA), and densitometry of each band was analyzed by the ImageJ software.

### CCK-8 assay

Cell Counting Kit-8 (Dojindo) was used in determining cell toxicity. Initially, A549, A549kd, A540oe cells and their control groups were seeded in 96-well plates at the concentration of 1 × 10^4^ cells (three wells per group), which was incubated in an incubator for 24 h. According to the protocol of first-line neoadjuvant chemotherapy (cisplatin 75 mg/m^2^ combined with pemetrexed 500 mg/m^2^), we mixed the two drugs as the same proportion to testify the drug efficiency in our in vitro cytotoxicity test. Mixed drugs at 0.125 μM -8 μM were added into a 96-well plate with cells. After 24 h, 10 μl of CCK-8 solution was added to each well for further incubation 2 h in 5% CO_2_ at 37 °C. The optical density (OD) was measured by a microplate reader at the 450 nm wavelengths. The cell viability rate was calculated as follow: (the OD value of experimental groups-the OD value of blank groups)/ (the OD value of controls- the OD value of blank groups).

### Drug intervention

According to CCK8 analysis, A549 cells, A549 with Ku80-silencing cells (A549kd), A549 with Ku80-oversxpression cells (A549oe), and the negative control (NCkd and NCoe) were treated with or without the mixed drugs (0.9 μM), respectively.

### Apoptosis detection by flow cytometry

Based on the manufacturer’s instructions, an Annexin V-PE/7AAD Apoptosis Detection Kit (BD Biosciences, Woburn, MA, USA) was used to test apoptosis. The cell apoptosis percentages were measured by a FACScan flow cytometer (BD Biosciences, Woburn, MA, USA).

### Statistical analysis

All date were analyzed by SPSS 19.0 computer software. The values are expressed as the means ± SD. Differences between two groups were analyzed by Student’s test. The relationship among the curative effect of neoadjuvant chemotherapy and Ku80 expression was analyzed by chi-square or Fisher’s exact test. *P* < 0.05 was defined as statistically significant.

## Results

### Ku80 correlated with resistance to neoadjuvant chemotherapy in patients with lung adenocarcinoma

**Fig. 1:**
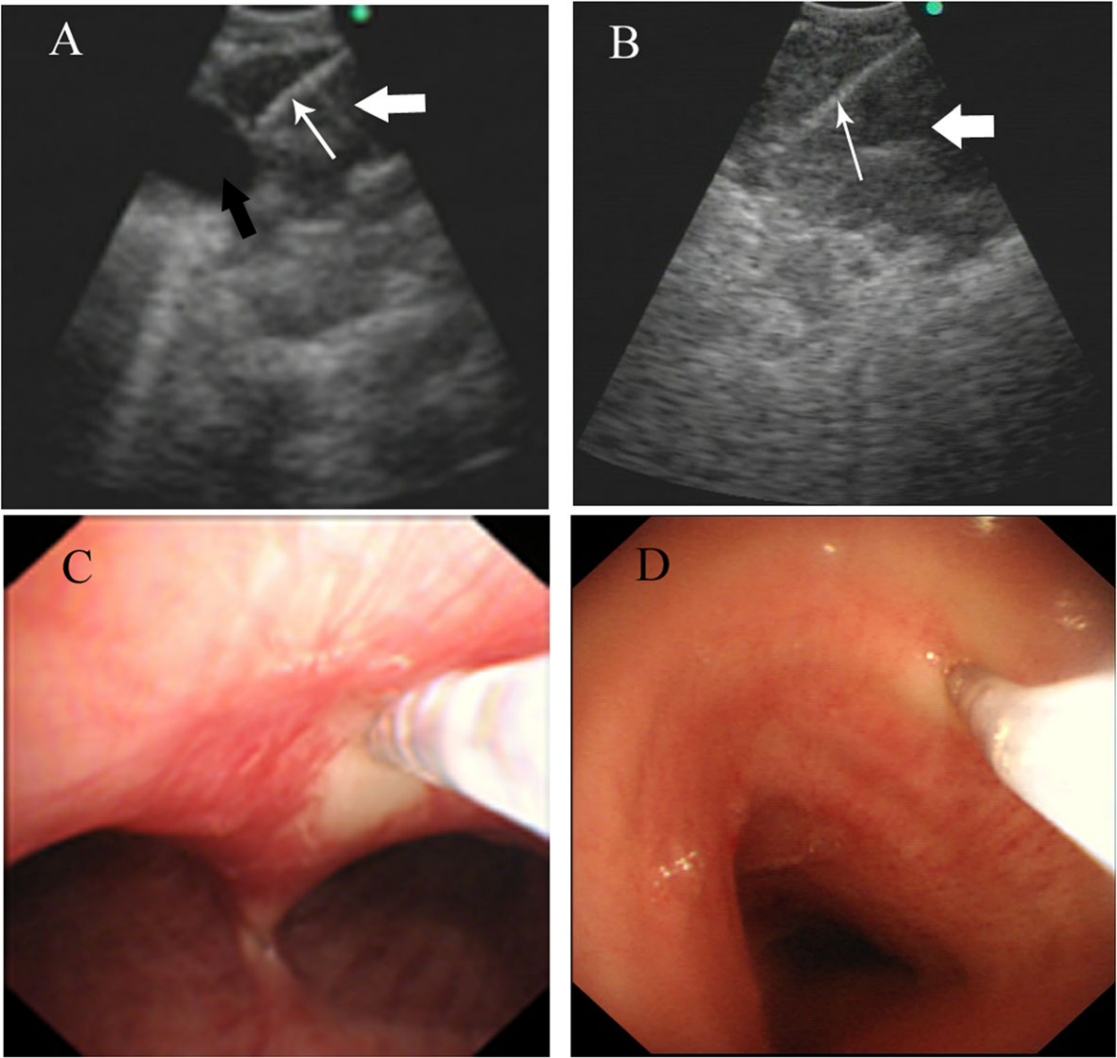
Endobronchial ultrasound-guided TBNA (EBUSTBNA). **a** The ultrasound image of lymph node biopsy. The needle (*white* slender arrow) was carefully inserted through bronchial wall to the enlarged lymph node (*white* thick arrow), avoiding injuring blood vessel (*black* arrow). **b** The fine needle (*slender* arrow) was inserted to enlarged lymph node (thick arrow). **c** and **d** are transbronchial needle aspiration (TBNA)

**Fig. 2:**
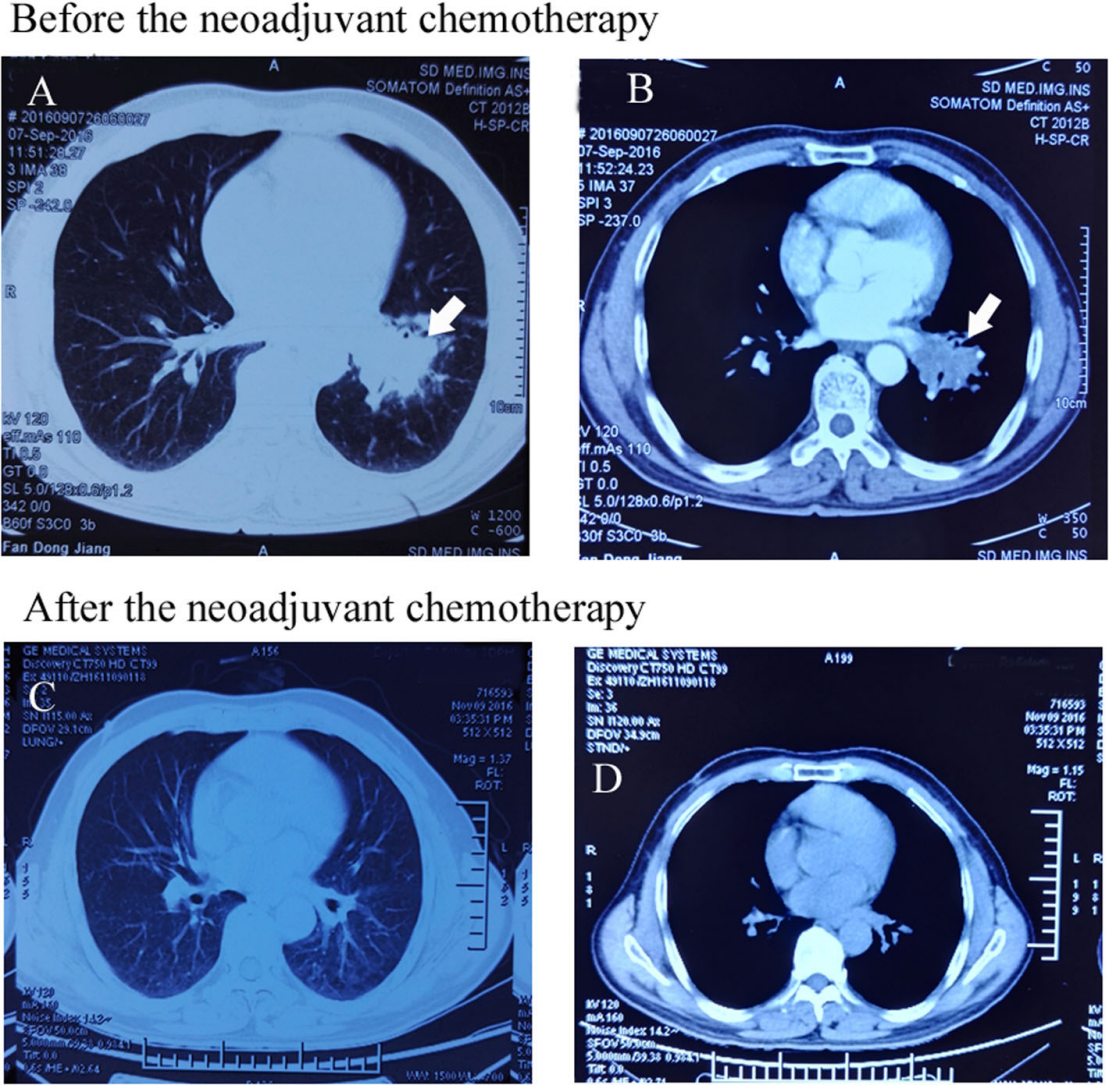
CT imaging before and after the neoadjuvant chemotherapy of lung adenocarcinoma in the response group. **a** CT transverse lung window imaging revealed the mass of left lung hilum (arrow). **b** In the mediastinum window, the mass showed heterogeneous enhancement, and the lesion invades left pulmonary vein (arrow). **c** and **d** are follow-up CT imaging after 2 months of neoadjuvant chemotherapy; the CT imaging showing the mass disappeared

**Fig. 3:**
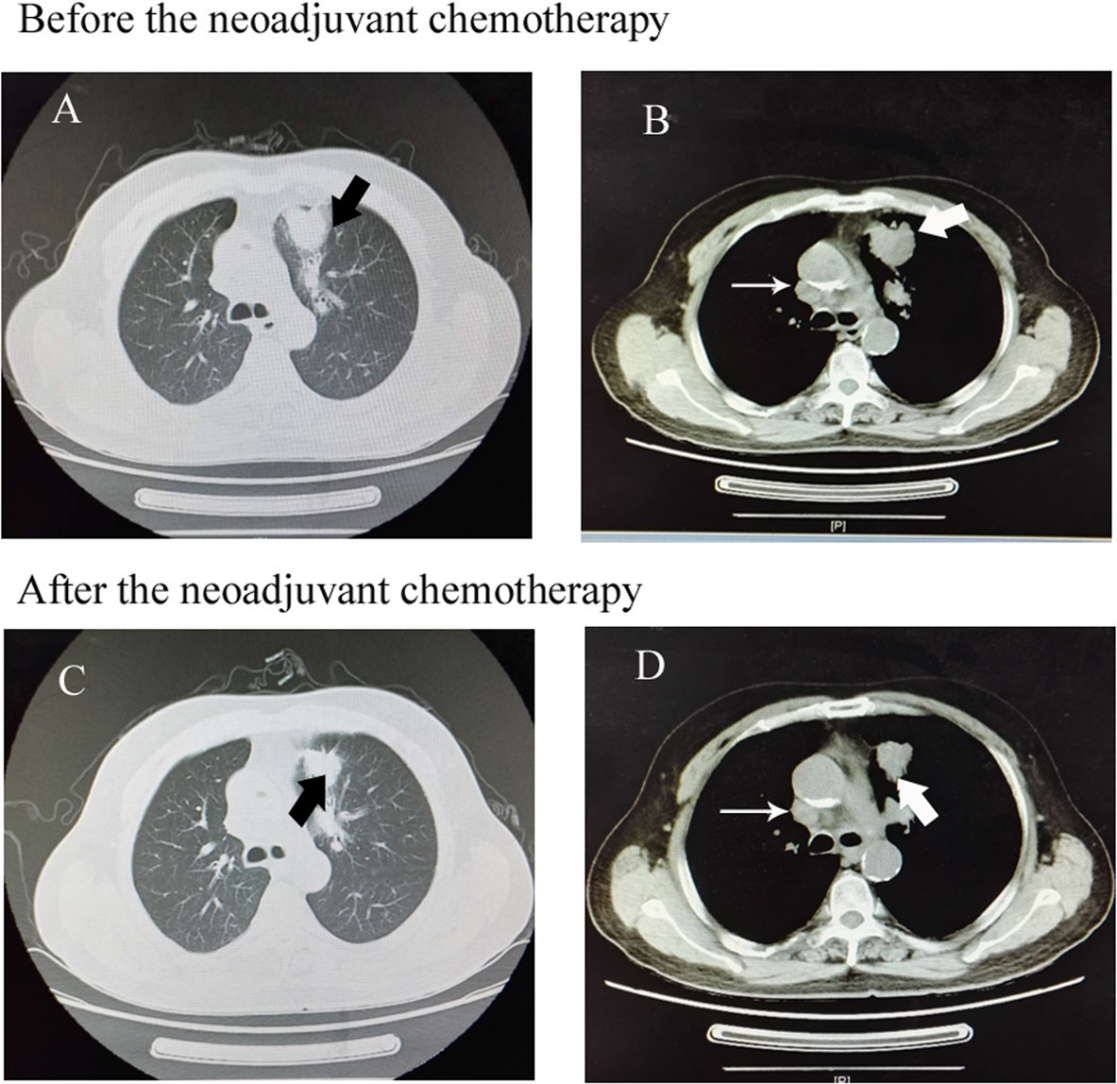
CT imaging before and after the neoadjuvant chemotherapy of lung adenocarcinoma in the nonresponse group. **a** and **b** are the CT imaging before the neoadjuvant chemotherapy of lung adenocarcinoma. **a** CT transverse lung window imaging revealed a nodule in the left upper lobe (arrow). **b** In the mediastinum window imaging showed lobulated and heterogeneously enhanced nodule (thick arrow) and metastasizes in tracheobronchial lymphnodes (slender arrow). **c** and **d** are follow-up CT imaging after 2 months neoadjuvant chemotherapy. The lesion was smaller (thick arrow), but the metastasized lymphnodes did not regress obviously (slender arrow)

**Fig. 4:**
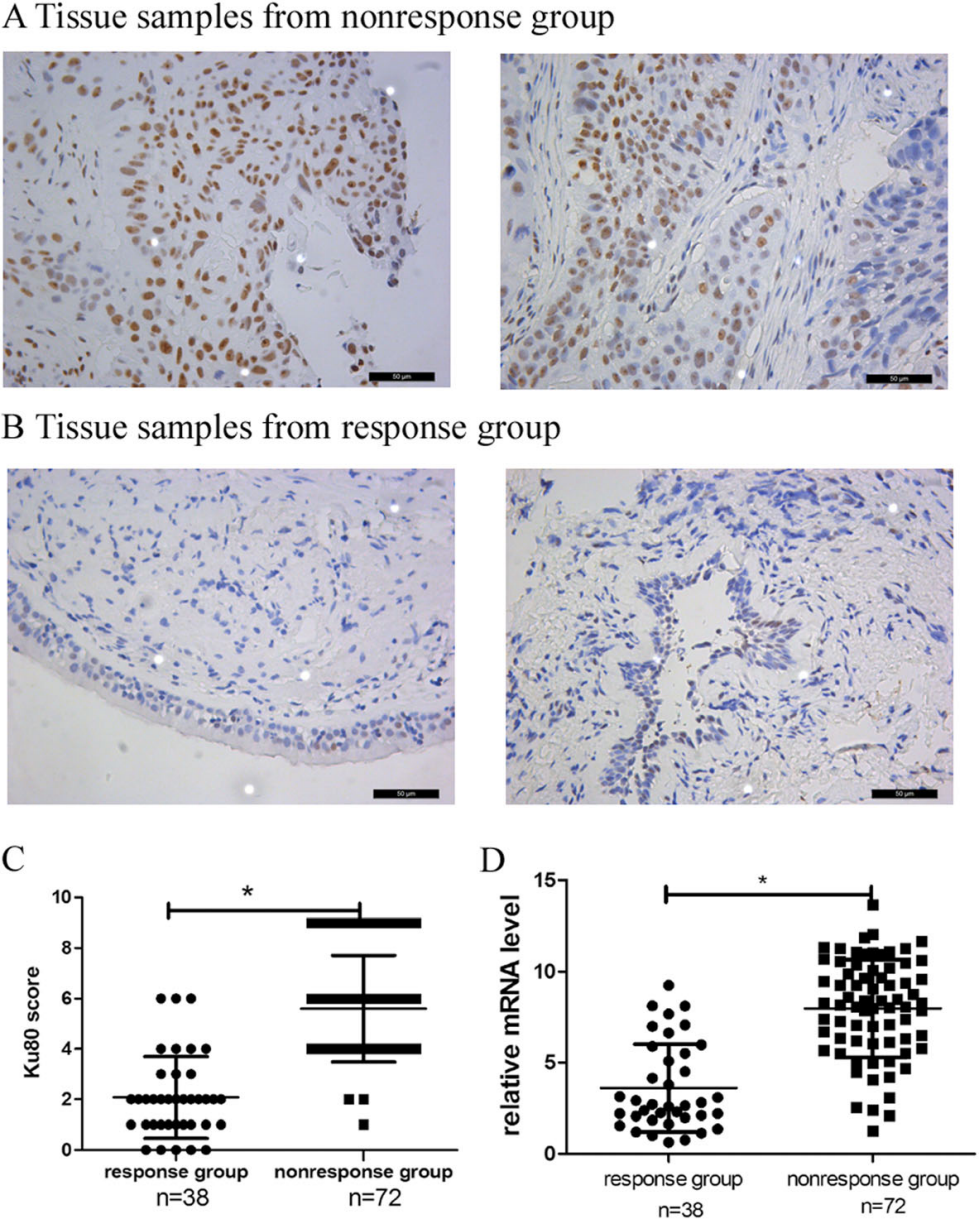
Ku80 protein and mRNA expression in lung cancer of the response and nonresponse groups. Ku80 protein expression in lung cancer of the response (**a**) and nonresponse groups (**b**) obtained by fiberoptic bronchoscopy. **c** Immunohistochemical scores of Ku80 were calculated in response group (*n* = 38) and nonresponse group (*n* = 72). Ku80 expression level of the response group was reduced compared to the nonresponse group (2.079 ± 1.617, 5.597 ± 2.114). **d** Quantitative RT-PCR analysis showed Ku80 mRNA expression between response (3.612 ± 2.392) and nonresponse (7.981 ± 2.684) groups. Data were shown as the mean ± SD. *p < 0.05

One hundred ten patients of clinical classification were stage IIIA and pathology diagnosed by bronchoscopy examination (Fig. 1). All patients received neoadjuvant chemotherapy based on cisplatin combined with pemetrexed for 2 cycles. 38 patients obtained obviously response to chemotherapy (Fig. 2), while the lesion or metastatic lymph node of other 72 patients did not decrease (Fig. 3) (Table 1). In the 38 cases of tumor tissues from the patients with response to chemotherapy, Ku80 was positive in 7 cases only (18.4%). Among 72 patients with no response to chemotherapy, Ku80 was positive in 69 cases (95.8%), which were significantly higher than Ku80 expressed in the response group. The IHS of Ku80 expression of response group to chemotherapy was 2.079 ± 1.617, while IHS of nonresponse group was 5.597 ± 2.114. The Ku80 expression in the response group was particularly lower compared to nonresponse group (*P* < 0.05, Fig. 4c). Subsequently, the differences between the response group and nonresponse group in the Ku80 mRNA level were observed. Compared with the nonresponse group, the response group had significantly lower level of Ku80 mRNA expression (*p* < 0.05, Fig. 4d). The relative mRNA level was 3.612 ± 2.392 in the response group and 7.981 ± 2.684 in the nonresponse group, respectively. Altogether, Ku80 protein and mRNA levels correlates with resistance to neoadjuvant chemotherapy in patients with lung adenocarcinoma.

**Table 1:** Ku80 expression of lung cancer detected by immunohistochemistry

Feature	patients (*n* = 110)	Ku80 protein level		p
		positive (*n* = 76)	negative (*n* = 34)	
Age at diagnosis				
≤ 60	66 (60%)	50 (65.8%)	16 (47.1%)	0.09
> 60	44 (40%)	26 (34.2%)	18 (52.9%)	
Gender				
Male	32 (29.1%)	24 (31.6%)	8 (23.5%)	0.50
Female	78 (70.9%)	52 (68.4%)	26 (76.5%)	
Smoking status				
Never	71 (64.5%)	50 (65.8%)	21 (61.8%)	0.68
Former or current smokers	39 (35.4)	26 (34.2)	13 (38.2%)	
Stage(T)				
T1-2	58 (52.7%)	30 (39.5%)	28 (82.4%)	0.00
T3-4	52 (47.2%)	46 (60.5%)	6 (17.6%)	
Lymph node metastasis				
N0-1	47 (42.7%)	22 (28.9%)	25 (73.5%)	0.00
N2	63 (57.2%)	54 (71.1%)	9 (26.5%)	
Response to chemotherapy				
(+)	38 (34.5%)	7 (9.2%)	31 (91.2%)	0.00
(-)	72 (65.5%)	69 (90.8%)	3 (8.8%)

### Lentiviral-mediated transfection of Ku80 shRNA and full length cDNA efficiently suppressed and upregulated Ku80 expression in A549 cells, respectively

**Fig. 5:**
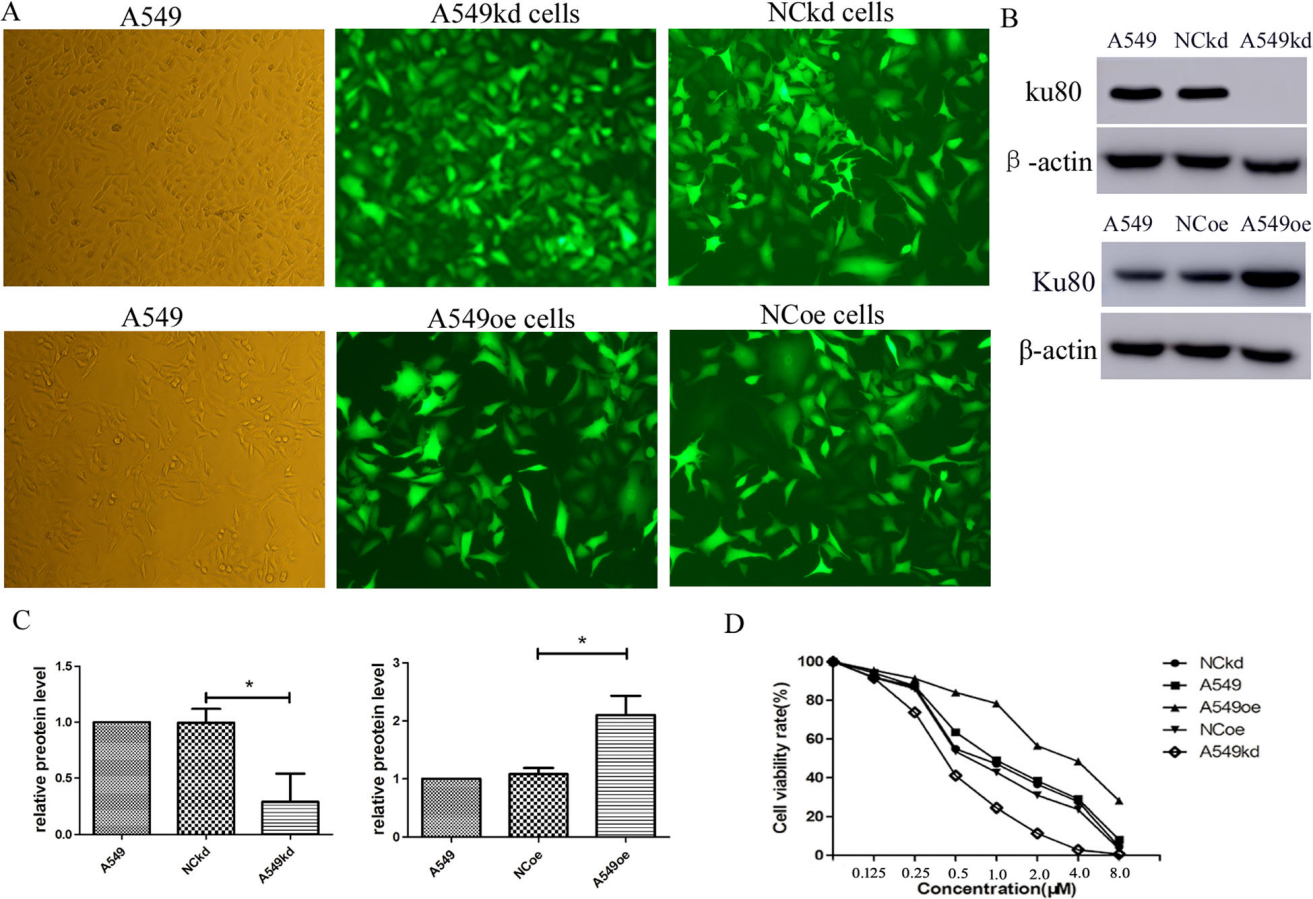
A549 cell transfection and cisplatin/pemetrexed treatment. **a** Normal A549 cell lines transfected by lentiviral vector. A549kd = A549 with Ku80-silencing cells. NCkd = A549 cells transfected by nonsilencing shRNA control vector. A549oe = A549 with Ku80-oversxpression cells. NCoe = A549 cells transfected by empty vector for over-expression. **b** The decreased and increased expression level of Ku80 in A549 cells transfected by Ku80 shRNA and cDNA, respectively. **c** Relative protein level of Ku80 as shown in (**b**). **d** A549 cells and transfected cells were treated with mixture of cisplatin and pemetrexed at concentration of 0, 0.125, 0.25, 0.5, 1, 2, 4, 8 μM for 24 h. Cell viability was performed using the CCK8 assay. Each experiment was performed in duplicate or triplicate. Data were shown as the mean ± SD. *p < 0.05

Cells were transfected with lentiviruses including specific shRNA (A549kd) and full length cDNA to manipulate Ku80 expression (A549oe), and transfected with corresponding nonsense sequence shRNA and empty vector as negative controls (NCkd and NCoe). To evaluate transfection efficacies of viral vectors, phase contrast image of fluorescence microscope was used. As shown in Fig. 5a, after transfection, GFP expression of transfected cells confirmed over 80%, indicating a high transduction efficiency. Western blot analysis showed that the expression of Ku80 was obviously knocked down and upregulated by Ku80 shRNA and full length cDNA, respectively (Fig. 5b and c). No significant difference was observed in the level of Ku80 expression among control lentiviral vector transfected and untransfected cells. These results illustrate that Ku80 cDNA and shRNA effectively manipulate the Ku80 gene expression in A549 cells.

### Effect of cisplatin combined with pemetrexed on A549 cell growth

In contrast to the control group, the mixed drugs reduced viability in A549 cells in a dose-dependent manner (Fig. 5d). The IC50 value of mixed drugs against A549 cells’ viability was 0.97 μM at 24 h. Hence, we chose 0.9 μM of mixed drugs to conduct further experiments to avoid the drug cytotoxicity. To confirm the role of Ku80 to predict the resistance to cisplatin combined with pemetrexed in adenocarcinoma, we used lenti-shRNA and lenti-cDNA to knock down and overexpress Ku80, respectively (Fig. 5a and b), then treated cells with different concentration of mixed drugs. Ku80-silencing A549 cells were 2.2- fold sensitive to mixed drugs (IC50 0.451 vs. 0.972) compared to untransfected A549 cells, which Ku80-overexpression A549 cells were 0.304- fold sensitive to mixed drugs (IC50 3.192 vs. 0.972) than untransfected A549 cells (*P* < 0.05, Fig. 5d) in terms of reduction of viability.

### Ku80 overexpression caused the resistance to A549 apoptosis induced by cisplatin combined with pemetrexed

**Fig. 6:**
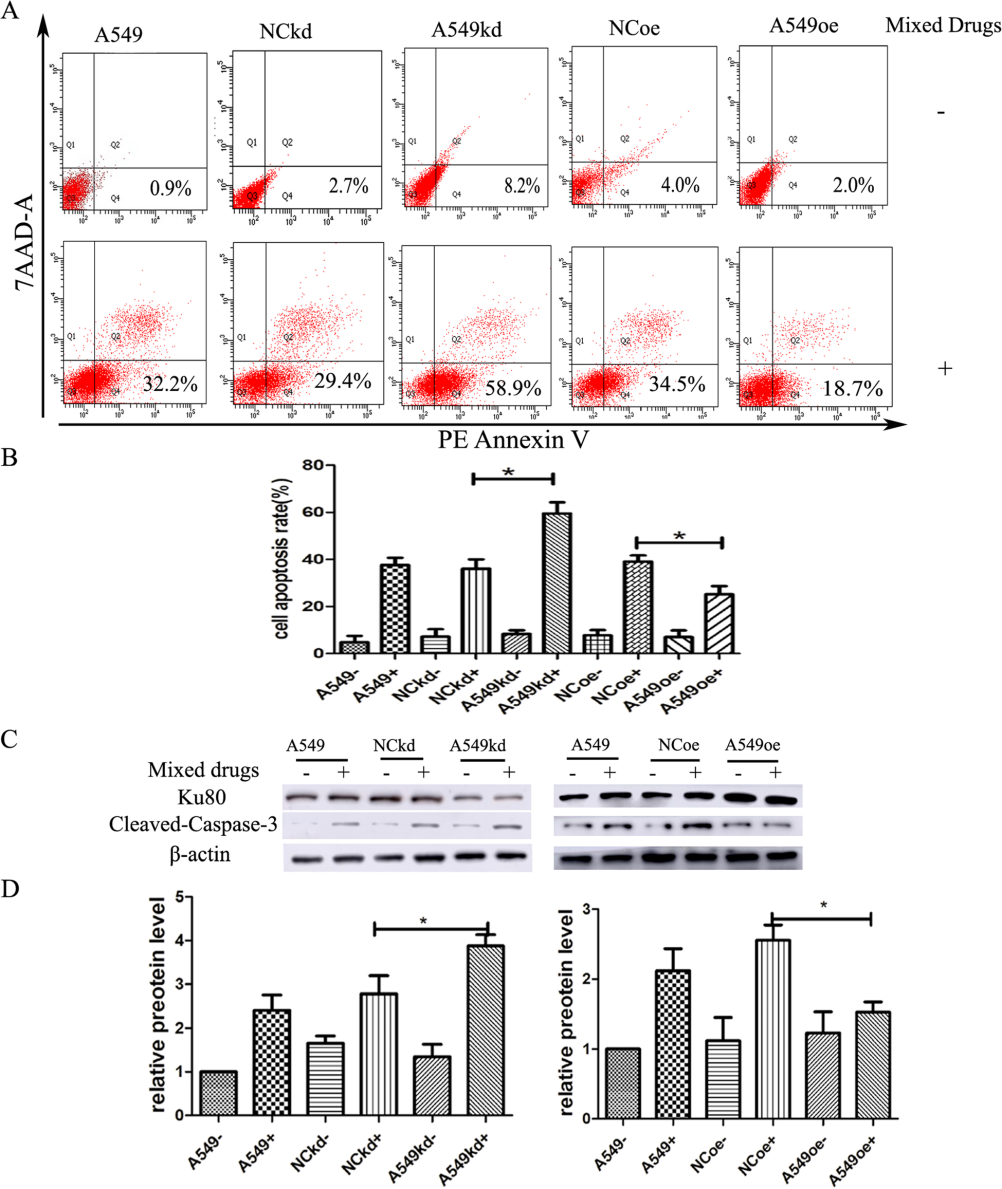
Ku80 overexpression caused the resistance to apoptosis induced by cisplatin/pemetrexed in A549 cells. **a** Flow cytometry apoptosis detection. A549 cells were transfected with shRNA and full length of cDNA to up- and down-regulate Ku80 expression, respectively. Cells were treated with 0.9 μM mixed drugs (cisplatin combined with pemetrexed) for 24 h. **b** Apoptosis rate was calculated in cells treated with the mixed drugs. **c** A549 cells and transfected cells were treated with the same concentrations of mixed drugs for 24 h, followed by Western blot for the detection of Ku80 and cleaved caspase-3 protein levels. **d** Quantification of Ku80 and cleaved caspase-3 levels as shown in (**c**). Data represented mean ± SD. Each experiment was performed in duplicate or triplicate. +: drug-treated groups; -: non-treated groups. *p < 0.05

Flow cytometry was used to identify whether Ku80 played a significant role in cell apoptosis caused by cisplatin combined with pemetrexed. Date from flow cytometry analysis displayed that A549 cells treated by mixed drugs underwent apoptosis, which was augmented in A549kd cells (58.9%). However, A549oe cells apoptosis was reduced compared to NCoe and normal A549 cells treated by the same drugs (18.7%) (*P* < 0.05, Fig. 6a and b). Furthermore, to confirm the relationship between apoptosis and Ku80 expression level, we detected the apoptosis associated protein cleaved caspase-3 by Western blotting. As shown in Fig. 6c and d, A549kd cells exhibited markedly higher levels of cleaved caspase-3 when treated with the mixed drugs, whereas cleaved caspase-3 level of A549oe cells revealed opposite changes. From the above, these results demonstrate that Ku80 causes lung adenocarcinoma cells resistance to apoptosis caused by cisplatin combined with pemetrexed.

## Discussion

Neoadjuvant chemotherapy has already been wildly accepted as a comprehensive therapeutic strategy, especially for local advanced lung cancer [12]. When most patients obtain accurate diagnosis, surgery may have not been as the first choice [3]. However, NSCLC cells developed resistance to chemotherapy. That is one of the main reasons that the patients get poor outcomes (5-year over survival < 10%) [13–15]. Furthermore, neoadjuvant chemotherapy was usually in experimental phases, and only a few patients get ideal effects [5]. For most patients with lymph node metastasis or with incompletely resection, chemotherapy is useless but increases the burden of patients.

An increasing number of researches indicate that there were lots of chemopredictive biomarkers such as DNA repair genes like ERCC1, β-tubulins or topoisomerases [16–18]. Ku80 as a key mediator of DNA DSB repair has been reported that its expression level could predict the prognosis and sensitivity to cisplatin [1, 19]. To determine this hypothesis in lung cancer, we used bronchoscopy to collect tumor tissue or metastatic lymphatic tissue for immunohistochemistry to measure the expression level of Ku80. After 2 cycles of chemotherapy, 38 patients responded effectively, which was reflected by reduced lesion diameters at least 30 percentages. In the response group, the numbers of Ku80-positive patients were much less than that in the nonresponse group. The IHS of Ku80 expression in the response group to chemotherapy was lower compared to the IHS in the nonresponse group. This clinical trial demonstrated that the expression level of Ku80 could predict the sensitivity of neoadjuvant chemotherapy based on cisplatin combined with pemetrexed.

Ku80 is involved in a mass of cellular process, including maintaining telomere, gene transcription and apoptosis resistance [6]. As an oncogene, Ku80 has been reported in lots of cancers such as bladder cancer, breast cancer, esophageal cancer and lung cancer [20–23]. These studies indicate that the repair capacity of DNA DSB plays an important role in modulating chemoresistance [1, 24]. In our study, we found that shRNA mediated knockdown of Ku80 in A549 cells promoted apoptosis induced by cisplatin combined with pemetrexed. In contrast, upregulated Ku80 expression caused resistance to cisplatin combined with pemetrexed. It has been shown that Ku80 could upregulate metalloproteinase (MMP)-2 and MMP-9 expression through activating ERK/JNK pathway [25], where MMPs correlate to angiogenesis, oncogenesis, tumor invasion and metastasis [26]. Furthermore, cells growth suppression and cycle arrest induced by Ku80 correlated to the p53 pathway [27]. Zun-yi Zhang et al. indicated a same conclusion that knocked down of Ku80 expression would enhance p53 and p21 expression, while restoration of Ku80 expression would reduce the expression level of p53 and p21 in A549 cells [28]. All these findings suggest that Ku80 might regulates MMPs, p53 and p21 as one of underlying mechanisms underlying the resistance to neoadjuvant chemotherapy, oncogenesis, tumorigenesis, metastasis and invasion in lung cancer, which needs further investigation.

## Conclusion

Our clinical study indicates that the chemoresistance to cisplatin combined with pemetrexed in patients with lung adenocarcinoma correlated with increased expression of Ku80 gene and protein. Overexpression of Ku80 attenuates, whereas Ku80 knockdown augments cisplatin/pemetrexed-induced apoptosis in A549 cells. Therefore, Ku80 levels could be a potential biomarker to predict the sensitivity of neoadjuvant chemotherapy or a therapeutic target via modulating apoptosis in lung adenocarcinoma.

## Abbreviations

A549kd: A549 with Ku80-silencing cells
A549oe: A549 with Ku80-oversxpression cells
CR: Complete Response
CT: Computed Tomography
DSBs: Double-strand breaks
ECL: Enhanced chemiluminescence
FBS: Fetal bovine serum
HRP: Horseradish peroxidase
MMPs: Metalloproteinases
NSCLC: Non-small cell lung cancer
OD: Optical density
PMSF: Phenylmethysulfonyl fluoride
PR: Partial Response
RPMI: Roswell Park Memorial Institute
